# Dutch food bank recipients have poorer dietary intakes than the general and low-socioeconomic status Dutch adult population

**DOI:** 10.1007/s00394-017-1540-x

**Published:** 2017-10-03

**Authors:** J. E. Neter, S. C. Dijkstra, A. L. M. Dekkers, M. C. Ocké, M. Visser, I. A. Brouwer

**Affiliations:** 1 Department of Health Sciences, Faculty Science, Vrije Universiteit Amsterdam, Amsterdam Public Health Research Institute, Amsterdam, The Netherlands; 2 National Institute for Public Health and the Environment, Bilthoven, The Netherlands; 3 Department of Internal Medicine, Nutrition and Dietetics, VU University Medical Center, Amsterdam, The Netherlands

**Keywords:** Nutrition, Dietary intake, Food assistance, Diet quality, Low-socioeconomic status, Public health, Dietary guidelines

## Abstract

**Purpose:**

Food-assistance program users are a specific group of nutritional concern, as they are often food insufficient and have poorer diet quality compared to non-food-assistance program users. The aim of our study was to assess dietary intake of Dutch food bank recipients (*n* = 167) and to compare this with dietary intake of a representative sample of the general population (Dutch National Food Consumption Survey (DNFCS-all): *n* = 1933), including a low-socioeconomic status (SES) sample (DNFCS-low SES: *n* = 312), using data from the DNFCS 2007–2010.

**Methods:**

In this cross-sectional study, 12 food banks throughout The Netherlands participated. Food bank recipients’ characteristics were assessed with a self-administered questionnaire. Dietary intake data were collected through three 24-h recalls. Habitual dietary intake (mean, percentiles, and 95% CI) was estimated for all samples. Differences between samples were determined by comparing the 95% CIs.

**Results:**

Mean age of the study population (62.9% female) was 48.6 years (SD:10.1). Mean energy intake was 1986 (95% CI 1830–2089) kcal. The majority of the Dutch food bank recipients had lower intakes than dietary reference intakes for dietary fiber, fruit, vegetables, and fish (range 86.6–99.3%), and a higher intake for saturated fat [88.1% (95% CI 84.1–98.9)]. Furthermore, mean intakes of energy, fiber, fruit, and vegetables were significantly lower in Dutch food bank recipients than in the DNFCS-all and the DNFCS-low-SES [e.g., daily mean fruit intake (g) food bank recipients 62.8 (95% CI 45.5–76.5), DNFCS-all 105.8 (95% CI 105.4–117.9), and DNFCS-low-SES 85.1 (95% CI 78.7–100.2)]. Fish intake was significantly lower compared with the DNFCS-all, but not compared with the DNFCS-low-SES.

**Conclusions:**

Dutch food bank recipients, who largely rely on the content of food parcels, are not able to meet the nutritional guidelines for a healthy diet, and their dietary intake is poorer than the general as well as the low-SES sample of the Dutch adult population. More research is needed on how to improve the dietary intake of this vulnerable population subgroup, by, e.g., revising the content of the food parcels, and to develop effective intervention activities.

## Introduction

Food-assistance program users are a specific group of people of nutritional concern, as they have limited resources to purchase food and largely rely on the availability and quality of donated food in the food parcels. We showed earlier [1] that the nutritional content of food parcels in The Netherlands is not in line with the dietary guidelines for a healthy diet. The provided amounts of energy, protein, and saturated fat for a single-person food parcel for a single day were higher than the nutritional guidelines, whereas the provided amounts of fruit and fish were lower. This previous work suggests that the nutritional guidelines for a healthy diet cannot be met if food supplied by food banks is the sole food source.

Food-assistance program users, i.e., eligible low-income families and individuals who make use of temporary food-assistance programs such as food banks, food pantries, and the supplemental nutrition-assistance program, are often food insufficient [2–6] and have poorer diet quality compared to non-food-assistance program users [7–9]. Studies on the nutritional intake of food-assistance program users show a lower consumption of fruit, vegetables, dairy products, or seafood compared with the guidelines [6, 7], the general population [10], or the low-socioeconomic status (SES) population [7, 11]. Furthermore, for vitamins A [12, 13], C, and E, and folate [12], fiber [6, 7, 12], calcium, and zinc [13], inadequate intakes have been reported. In addition, one study revealed that food-assistance program users, compared to the general population, show higher prevalences of folate and vitamin D deficiency, and of low vitamin C, ß-carotene, and zinc levels [10]. The inadequate nutritional intake and suboptimal nutritional status may lead to malnutrition and higher risks of nutrition-related chronic diseases [12, 14].

The Dutch Food Bank is a charitable non-governmental organization which collects and distributes donated foods through 167 food banks with 535 distribution points throughout The Netherlands. In 2016, the food banks supported approximately 135 thousand individuals, and they aimed to provide food parcels weekly that supplement the normal diet for 2–3 days, up to a maximum of 3 years [15]. The criteria to become a food bank recipient were that individuals living alone with a monthly disposable income < 180 Euros qualified for food assistance as did families with a monthly disposable income of < 180 Euros with the additional income allowance of 60 Euros per adult and 50 Euros per child (< 18 years of age). The content of food parcels largely depends on donated foods by food companies, supermarkets, and individuals, and consequently varies by week and by food bank.

Worldwide, food-assistance programs significantly differ in the way the food is collected and provided. Three studies assessing the nutritional intake of adult food-assistance program users were conducted in the USA [6, 7, 12], two in Canada [10, 13], and two in Europe: France [10] and Germany [11]. Dutch data are not available yet. Insight in the nutritional intake of Dutch food bank recipients is important to determine whether food bank recipients are able to meet the nutritional guidelines for a healthy diet, given that the provided food parcels do not meet these guidelines [1]. This study takes into account foods eaten from the food parcel as well as additionally bought foods, and provides a more accurate assessment of dietary sufficiency of food-assistance users than merely analyzing the content of the food parcels. This knowledge is necessary to improve the nutritional quality of food bank parcels, which is likely to positively impact the dietary intake of this vulnerable population subgroup and to reduce their risk of nutrition-related diseases.

Therefore, the aims of this study were (1) to assess the dietary intake of Dutch food bank recipients, (2) to compare the dietary intake of Dutch food bank recipients with dietary reference intakes (DRIs), and (3) to compare the dietary intake of Dutch food bank recipients with the dietary intake of a representative sample of the general and low-SES Dutch adult population.

## Materials and methods

In The Netherlands, there were approximately 135 food banks in 2011–2012. The inclusion criterion for food banks to participate in our study was provision of food parcels once a week. The ones providing food parcels every other week were excluded. To obtain a sample which reflects the different food banks and, therefore, recipients best, we recruited food banks that varied in the number of recipients (smaller [*n* < 50], medium [*n* = 50–100] as well as larger [*n* > 100] numbers of recipients), urbanization level (smaller [e.g., 30,000 inhabitants] as well as larger [e.g., > 120,000 inhabitants] cities), and region (different regions across The Netherlands). Eventually, food banks were selected based on their willingness to participate. Each participating food bank had its own way of working. They either collect foods and compose the content of the food parcels themselves, or they receive ready-to-supply food parcels, which are composed elsewhere (e.g., a distribution centre, another food bank). However, they all aim to supply food parcels of which the content is—more or less—similar.

The study was approved by the Medical Ethical Committee of the VU Medical Center in Amsterdam, The Netherlands, as well as the national board of the Dutch Food Bank. Participants were exempt from informed consent by the Medical Ethical Committee of the VU Medical Center. Prior to this study, a pilot was carried out among four food bank recipients from the food bank in Huizen, to test our materials and the feasibility of the measurements. This was done by administering the general and food security questionnaires and conducting the 24-hour (24-h) recall interview and anthropometric measurements in the four food bank recipients to find out whether the questions in the questionnaires were clear, and how much time it would take to conduct the measurements and interviews.

### Study sample

The target population consisted of recipients of the 12 participating food banks. We recruited recipients through information letters, active recruitment at the food banks, and promotional posters. They could sign up for the study with an application form, by telephone or e-mail within either 2 or 3 weeks after they were informed about the study through information letters, the researchers at the food banks, and promotional posters. Inclusion criteria for participation were: (1) ≥ 18 years of age; (2) adequate command of the Dutch language to participate in oral and written interviews; (3) recipient of a Dutch food bank > 1 month; (4) collect own food parcel at the food bank; and (5) possible to be contacted by phone. Only one member per household was enrolled. Of the approximately 1200 food bank recipients at the participating food banks, 284 voluntarily indicated that they were interested to participate, of which 173 (60.9%) actually participated in the study. For 66 of the 111 recipients who signed up for participation, but did not participate, we were able to ascertain the reason for non-participation: (1) not a food bank recipient anymore at the start of the study (*n* = 19); (2) lack of time (*n* = 12); (3) no longer wants to participate (*n* = 11); (4) no adequate command of the Dutch language (*n* = 8); and (5) other reasons (*n* = 16) such as illness or not willing to participate in anthropometric measurements. Of the 45 remaining recipients who did not participate after signing up, 15 recipients did not show up at the first measurements, 29 recipients did not respond to e-mail and/or phone calls, and 1 recipient did not fill in contact information. Data collection was scheduled between September 2011 and February 2012, and collected through a general questionnaire, anthropometric measurements, and 24-h recalls. Participants were excluded from data analyses in case of < 3 24-h recalls and/or a missing general questionnaire (*n* = 6). A total of 167 participants, approximately 14% of the 1200 food bank recipients at the participating food banks, were included for data analyses. Participants who completed the study received both a gift coupon of 5 Euros and a small incentive.

### Questionnaires

Participants completed a self-administered general questionnaire at the food bank, which consisted of questions regarding socio-demographics, lifestyle factors, nutrition, and the appreciation of the food parcels. Participants who had difficulties in reading or writing, were offered help filling in the questionnaire.

Socio-demographics included date of birth, sex, duration of being recipient of the Dutch food bank (0–6, 6–12 months, and > 12 months), household composition (number of children < 18 years, ≥ 18 years, and adults in household), and educational level. For the covariable household composition, we created three categories: single parent household (including one adult and at least one child), household without children (including at least one adult and no children), and multiple household with children (including at least two adults and at least one child). We created three levels of education: low (less than finished elementary school), medium (finished elementary school) and high (higher than finished elementary school).

Lifestyle factors included current smoking (yes/no) and physical activity. Physical activity was established by asking “How many days a week are you physically active at moderate intensity for at least 30 min?”. Moderately intense physical activity included sport activities, walking, cycling, gardening, and performing heavy housework. We created two categories (0–4 days a week/5–7 days a week) based on the national physical activity guidelines for adults; at least 5 days a week 30 min of physical activity at moderate intensity [16].

Regarding nutrition, we asked participants “How satisfied are you with your current food intake?” (very unsatisfied, unsatisfied, not unsatisfied/not satisfied, satisfied, and very satisfied), and “How healthy do you think your current food intake is?” (very unhealthy, unhealthy, not unhealthy/not healthy, healthy, and very healthy). Questions regarding the food parcels included: “How satisfied are you usually with the content of the food parcel?” (very unsatisfied, unsatisfied, not unsatisfied/not satisfied, satisfied, and very satisfied), “How healthy do you think the content of the food parcel is in general?” (very unhealthy, unhealthy, not unhealthy/not healthy, healthy, and very healthy).

### Anthropometric measurements

Trained researchers measured participants body height, body weight, and waist circumference, according to a standardized protocol developed for this study. For the hereafter-described height and weight measurements, participants were asked to remove any items from their pockets and to take off their shoes and coat. A portable stadiometer, type Seca ^®^ 214, was used to measure height to the nearest 0.1 cm, and a calibrated mechanical scale, type Seca ^®^ 761, to measure weight to the nearest 0.5 kg. Waist circumference was measured in duplicate on bared skin, with a measurement tape to the nearest 0.1 cm. Body mass index (BMI) was calculated as measured weight (kg) divided by measured height squared (m^2^). BMI cut-off points of the WHO were used to define weight status [17].

### Data collection of the 24-h recalls

Data on food intake and supplement use were collected by trained interviewers through three 24-h recalls in a 3-week period using the USDA five-step multiple pass method (MPM) [18–20]. The MPM method was developed for collecting interviewer-administered 24-h recalls and includes multiple passes through 24 h of the previous day, during which respondents receive cues to help them remember and describe foods and drinks that they consumed [18–20].

The first 24-h recall was conducted in-person, during which a table scale, type KERN FCE 6 K2 ^®^, extensive tableware, and a portion-size photo book were used to assist in portion-size estimation of consumed foods and drinks. The portion-size photo booklet was taken home by the participants to use in the second and third 24-h recalls, which were conducted by phone. We aimed to obtain dietary information on two different weekdays and one weekend day, or if not possible, on three different weekdays. Twenty-four hour recalls were conducted at dates and times unannounced to the participants.

### Data processing of the 24-h recalls

All recorded foods and drinks from the three 24-h recalls were coded with the corresponding Dutch Food Composition Table code (NEVO-code) [21, 22]. Portion sizes consumed were entered in gram weights. Energy content and nutrient composition of the food and drinks consumed was determined using the 2010 NEVO-database [22], which provides the nutrient composition of foods and drinks commonly consumed in The Netherlands.

### Data Dutch National Food Consumption Survey 2007–2010

To compare dietary intake of the Dutch food bank recipients with dietary intake of a representative sample of the general Dutch adult population, we used a selection of the food consumption data from male and female adult participants of the Dutch National Food Consumption Survey 2007–2010 (DNFCS) [23]. This selection of the DNFCS consisted of participants aged 23–69 years, similar to the age range of the Dutch food bank recipients, and included a low-SES sample, based on the highest completed educational level; primary or lower vocational education. DNFCS-all indicates all adults aged 23–69 years (*n* = 1933), whereas DNFCS-low-SES indicates the subgroup (*n* = 312) with a low-socioeconomic status (SES). Briefly, participants were drawn from representative consumer panels. Inclusion criteria were a maximum of one person per household and an adequate command of the Dutch language. Data were collected between March 2007 and April 2010. Participants completed a general questionnaire either on paper or online on various background and lifestyle factors [23]. Two non-consecutive 24-h recalls using the EpicSoft software [24] were conducted per participant by telephone, at dates and times unannounced to the participants [23]. The 2011 NEVO database was used to determine energy content and nutrient intake. In addition, self-reported height (cm) and body weight (kg) were recorded during the telephone interviews. All interviews were carried out by trained dieticians.

### Statistical analyses

Characteristics of the food bank recipients and the DNFCS participants were analyzed with IBM SPSS Statistics for Windows version 21.0 (Armonk, NY: IBM Corp, USA). Descriptive statistics were used to summarize the participant characteristics. Continuous variables were presented as mean and standard deviation (SD), whereas categorical variables were presented as frequency and relative frequency.

To make the dietary intake data more normal distribution like, a Box–Cox transformation was used. This was visually tested with Q–Q plots. Habitual dietary intake data, i.e., the long-term mean intake of energy, macronutrients, fruit, vegetables, and fish with the accompanying distributions, were estimated from the observed daily intake by correction for the intra-individual (day-to-day) variation. This was done using SPADE (Statistical Program to Assess Dietary Exposure, version 3.1, RIVM), which was implemented in R [25] version 3.3.1 (The R Foundation for Statistical Computing, Austria). This is a highly advanced method developed by the National Institute for Public Health and the Environment to estimate habitual dietary intake distributions on national level [26], which takes the within- and between-person variation into account. Uncertainty in the habitual intake distribution and the proportion below or above a cut-off value was quantified with bootstrap (1000 samples), providing 95% confidence intervals (95% CIs). Analyses for DNFCS-all and DNFCS-low-SES included age and sex as covariables to adjust for possible differences in dietary intake between males and females, and were weighed for small deviations in sociodemographic characteristics (level of education, region, and urbanization) from the Dutch population, deviations from an equal distribution of days of the week, and season of data collection based on the first interview day [23].

For the analyses of the habitual dietary intake of Dutch food bank recipients, an extension of SPADE was used to include non-personal covariables, i.e., household composition, and the number of days between receiving a food parcel and the day of the recall, besides age and sex. These non-personal covariables were added to the model, because they are possibly associated with dietary intake; adult caregivers may sacrifice their own diet to avoid that their children should experience hunger [27], and the more days between receiving a food parcel and the day of the recall, the less food is assumed to be available from the food parcel. Furthermore, a weight factor was included for sex (i.e., male: 1.34756 and female: 0.79477) to make the results more comparable with the DNFCS, where the proportion of males and females was nearly 50/50. The estimated habitual intake distribution is presented by its mean and some percentiles with the corresponding 95% CI.

To evaluate the dietary intake, energy, macronutrients, fruit, vegetables, and fish intakes were compared with DRIs, if available. The contribution of nutrients from dietary supplements was not considered. We compared dietary intake with the Dutch nutritional guidelines for a healthy diet which were in use at the time we collected our data [28–30]. The percentages of participants below or above these DRIs [28–33] were based on cut-off values of the recommended dietary allowance for carbohydrates, and protein, the daily adequate intake for fat, the daily tolerable upper level for polyunsaturated fat, and trans-fat, and the daily recommendation for fiber, fruit, vegetables, and fish.

We determined differences in estimated habitual intakes and percentages of participants not meeting the DRIs between the Dutch food bank recipients, the DNFCS-all and the DNFCS-low-SES by comparing the 95% CIs. When the 95% CIs did not overlap, the difference between the groups was considered to be statistically significant.

## Results

### Food banks

The 12 participating food banks (with number of participants indicated in brackets) from 8 provinces were located in two smaller (Boxtel [*n* = 8], Zeewolde [*n* = 5]), four medium (Alkmaar [*n* = 31], Delft [*n* = 9], Hilversum [*n* = 10], and Wageningen [*n* = 5]), and six larger size (Amersfoort [*n* = 16], Apeldoorn [*n* = 8], Breda [*n* = 7], Enschede [*n* = 46], Groningen [*n* = 10], and Rotterdam [*n* = 18]) cities. This included one small food bank (Zeewolde), five medium size food banks (Boxtel, Delft, Hilversum, Rotterdam, and Wageningen), and six large food banks (Alkmaar, Amersfoort, Apeldoorn, Breda, Enschede, and Groningen).

### Dutch food bank recipients

Complete data were collected from 167 Dutch food bank recipients, of whom 62.9% were female (Table 1). Mean age of the total study sample was 48.6 (SD: 10.1) years and the majority of the participants was low educated (66.5%). Furthermore, 58.1% were current smokers and 61.1% reported to meet the national physical activity guideline. More than two-third of the participants were either overweight or obese. The majority of the participants (60.5%) were usually either satisfied or very satisfied with content of the food parcel, and 50.9% perceived the content of the food parcel either as healthy or as very healthy.

**Table 1:** Characteristics of 167 Dutch food bank recipients measured in 2011/2012, the DNFCS-all (*n* = 1933), and the DNFCS-low-SES (*n* = 312), measured between 2007 and 2010

Characteristics	Food bank, *n* = 167	DNFCS-all, *n* = 1933	DNFCS-low-SES, *n* = 312
Age, years	48.6 ± 10.1	44.0 ± 14.2	49.3 ± 14.3
Sex			
Male	62 (37.1)	964 (49.9)	158 (50.6)
Female	105 (62.9)	969 (50.1)	154 (49.4)
Duration of being recipient (months)			
0–6	44 (26.3)		
6–12	44 (26.3)		
≥ 12	79 (47.3)		
Household composition			
Single parent household	38 (22.8)		
Household without children	100 (59.9)		
Multiple household with children^a^	29 (17.4)		
Educational level			
Low; less than finished elementary school	111 (66.5)		
Medium; elementary school	36 (21.6)		
High; higher than elementary school	20 (12.0)		
Current smoking			
Yes	97 (58.1)	504 (26.1)	91 (29.2)
No	70 (41.9)	1428 (73.9)	221 (70.8)
BMI^b^, kg/m^2^	28.9 ± 6.5	26.2 ± 4.9	27.1 ± 4.5
Weight status (kg/m^2^)			
Underweight; BMI < 18.5	3 (1.8)	35 (1.8)	1 (0.3)
Normal weight; BMI 18.5–24.9	47 (28.5)	837 (43.3)	108 (34.6)
Overweight; BMI 25–29.9	54 (32.7)	700 (36.2)	134 (42.9)
Obese; BMI ≥ 30	61 (37.0)	360 (18.6)	69 (22.1)
Waist circumference, cm			
Men	101.8 ± 16.8		
Women	102.0 ± 16.7		
Physically active ≥ 30 min/day^c^			
0–4 days/week	65 (38.9)	634 (32.8)	99 (31.7)
5–7 days/week	102 (61.1)	1290 (66.7)	207 (66.4)
Unknown	–	9 (4.7)	6 (1.9)
Satisfaction with current food intake			
Very unsatisfied	5 (3.0)		
Unsatisfied	21 (12.6)		
Not unsatisfied/not satisfied	47 (28.1)		
Satisfied	87 (52.1)		
Very satisfied	7 (4.2)		
Perceived healthiness current food intake			
Very unhealthy	4 (2.4)		
Unhealthy	18 (10.8)		
Not unhealthy/not healthy	65 (38.9)		
Healthy	74 (44.3)		
Very healthy	6 (3.6)		
Satisfaction with food parcel			
Very unsatisfied	7 (4.2)		
Unsatisfied	9 (5.4)		
Not unsatisfied/not satisfied	50 (29.9)		
Satisfied	79 (47.3)		
Very satisfied	22 (13.2)		
Perceived healthiness food parcel			
Very unhealthy	5 (3.0)		
Unhealthy	18 (10.8)		
Not unhealthy/not healthy	59 (35.3)		
Healthy	81 (48.5)		
Very healthy	4 (2.4)		

DNFCS-all: data from the Dutch National Food Consumption Survey 2007–2010 among 1933 adults in the age range 23–69 years

DNFCS-low-SES: data from the Dutch National Food Consumption Survey 2007–2010 among 312 low educated adults in the age range 23–69 years

Values are presented as mean ± SD, as frequency with between brackets the relative frequency as a percentage

Total *n* = 167 (Duch food bank). BMI, weight status *n* = 165; Waist circumference n = 163. Two 10-week pregnant female participants were excluded

Total *n* = 1933 (DNFCS-all). BMI, smoking *n* = 1932

^a^Households with children and more than one adult

^b^Food bank recipients: measured; DNFCS-all and DNFCS-low-SES: self-reported

^c^Food bank recipients: moderate-intense physical activity; DNFCS-all and DNFCS-low-SES: strenuous physical activity

### DNFCS-all and DNFCS-low-SES samples

Mean age of the DNFCS-all (*n* = 1933) and DNFCS-low-SES (*n* = 312) was 44.0 (SD: 14.2) and 49.3 (SD: 14.3) years, respectively (Table 1). Males and females were about equally represented in both samples. In the DNFCS-all, 26.1% was a current smoker, 66.7% reported to meet the national physical activity guideline, and 54.8% was either overweight or obese. In the DNFCS-low-SES, 29.2% was a current smoker, 66.4% reported to meet the national physical activity guideline, and 65.0% was either overweight or obese.

### Comparison of Dutch food bank recipient dietary intake with DRIs

Mean daily habitual intakes (percentiles and 95% CIs) of energy, macronutrients, fruit, vegetables, and fish are presented in Table 2. The mean habitual energy intake of Dutch food bank recipients was 1986 (1830–2089) kcal (Table 2). Intake of saturated fat [13.4 (12.3–14.2) en%] was higher and intake of dietary fiber [16.5 (14.9–17.4) g] was lower than the dietary guidelines for a healthy diet.

**Table 2:** Mean daily habitual intakes (percentiles and 95% CIs) of energy, macronutrients, fruit, vegetables, and fish of 167 Dutch food bank recipients, the DNFCS-all (*n* = 1933), and the DNFCS-low-SES (*n* = 312)

	Sample	Mean habitual intake (95% CI)	P5	P25	P50	P75	P95
Energy (kcal)	Food bank	1986 (1830–2089)^a,b^	782	1334	1850	2499	3625
	DNFCS-all	2274 (2249–2345)	1419	1862	2235	2644	3260
	DNFCS-low-SES	2364 (2310–2442)	1523	1919	2329	2770	3340
Carbohydrates total (en%)	Food bank	47.8 (46.0–48.6)^a,b^	36.5	43	47.6	52.7	59.3
	DNFCS-all	43.7 (43.4–44.1)	34.5	39.9	43.7	47.5	53
	DNFCS-low-SES	43.5 (42.6–44.1)	34.7	39.8	43.4	47.0	52.3
Mono- and disaccharides (en%)	Food bank	21.4 (20.1–22.5)	9.8	15.6	20.5	26.5	35.5
	DNFCS-all	19.8 (19.5–20.1)	11.4	15.9	19.5	23.3	29.4
	DNFCS-low-SES	19.7 (18.9–20.4)	11.3	15.7	19.2	23.1	29.4
Polysaccharides (en%)	Food bank	27.3 (25.4–30.1)^a,b^	16.5	22.4	26.9	32.1	39.2
	DNFCS-all	23.9 (23.7–24.2)	18	21.4	23.8	26.3	29.8
	DNFCS-low-SES	23.7 (23.2–24.3)	18.7	21.7	23.7	25.8	28.7
Protein total (en%)	Food bank	15.2 (14.6–15.6)	11.2	13.4	15.1	17	19.5
	DNFCS-all	15.7 (15.5–15.9)	11.9	13.9	15.5	17.2	20.0
	DNFCS-low-SES	15.5 (15.2–15.8)	11.9	13.8	15.3	16.9	19.5
Fat total (en%)	Food bank	36.2 (33.1–39.4)	26.8	32.3	36.0	40.2	45.4
	DNFCS-all	34.2 (33.9–34.6)	27.3	31.3	34.1	37	41.1
	DNFCS-low-SES	35.4 (34.8–36.1)	28.4	32.7	35.5	38.3	42.1
Fat monounsaturated (en%)	Food bank	12.1 (11.6–13)	8.4	10.5	12	13.7	15.9
	DNFCS-all	11.7 (11.6–12.9)	9	10.5	11.7	12.9	14.6
	DNFCS-low-SES	12.2 (11.8–12.4)	9.4	11	12.1	13.3	15.1
Fat polyunsaturated (en%)	Food bank	6.8 (6.2–7.3)	4.0	5.4	6.6	7.9	10.2
	DNFCS-all	7.0 (6.6–7.2)	4.9	6.0	6.9	7.9	9.5
	DNFCS-low-SES	7.1 (6.8–7.3)	5.1	6.2	7.0	8	9.4
Fat saturated (en%)	Food bank	13.4 (12.3–14.2)	8.7	11.4	13.3	15.4	18.1
	DNFCS-all	13 (12.8–13.6)	9.4	11.5	12.9	14.4	16.7
	DNFCS-low-SES	13.1 (12.8–13.5)	9.5	11.5	13.1	14.6	17
Fat trans (en%)	Food bank	0.66 (0.59–0.73)^a,b^	0.27	0.45	0.61	0.82	1.17
	DNFCS-all	0.58 (0.57–0.59)	0.34	0.46	0.56	0.68	0.88
	DNFCS-low-SES	0.56 (0.53–0.59)	0.31	0.44	0.54	0.67	0.88
Alcohol^c^ (g)	Food bank	7.6 (5–10.1)^a,b^	0.0	0.2	1.3	8.8	37.5
	DNFCS-all	14.2 (13.1–15.2)	0.0	1.9	8.7	21.2	46.9
	DNFCS-low-SES	12.6 (10.6–15.2)	0.0	0.9	6.5	19.1	44.9
Dietary fiber (g)	Food bank	16.5 (14.9–17.4)^a, b^	6.9	11.5	15.4	20.7	28.9
	DNFCS-all	20.8 (20.5–21.7)	12.4	16.8	20.4	24.3	30.6
	DNFCS-low-SES	19.9 (19.6–20.8)	12.4	16.3	19.5	23.1	29
Fruit (g)	Food bank	62.8 (45.5–76.5)^a,b^	4.2	19.7	48.3	91.9	170.6
	DNFCS-all	105.8 (105.4–117.9)	14.9	50.6	91.8	146.3	244.5
	DNFCS-low-SES	85.1 (78.7–100.2)	14.1	42.8	75.1	116.8	190.6
Vegetables (g)	Food bank	123.0 (94.8–134)	32.9	71.6	111.0	161.3	254.3
	DNFCS-all	128.5 (127.3–137.0)	65.7	97.0	123.7	154.8	207.3
	DNFCS-low-SES	109.1 (104.8–125.2)	52.0	80.1	104.4	133	182.5
Fish (g)	Food bank	8.5 (5.4–12.9)^a^	1.1	3.1	6.4	11.9	23.3
	DNFCS-all	17.7 (15.8–19.4)	1.4	5.6	12.1	23.5	53
	DNFCS-low-SES	14.0 (11–18.7)	1.3	4.5	9.5	18.4	41.9

DNFCS-all: data from the Dutch National Food Consumption Survey 2007–2010 among 1933 adults in the age range 23–69 years

DNFCS-low-SES: data from the Dutch National Food Consumption Survey 2007–2010 among 312 low educated adults in the age range 23–69 years

Dutch food bank: Adjusted for age, sex, household composition, and number of days between receiving a food parcel and the day of the recall

DNFCS-all and DNFCS-low-SES: Adjusted for age and sex

*en%*  energy percentage

^a^Significantly different from the DNFCS-all, determined by comparing the 95% CIs

^b^Significantly different from the DNFCS-low-SES, determined by comparing the 95% CIs

^c^Assuming that one glass of alcohol contains 10 g of alcohol

Table 3 presents the percentages (95% CIs) for not meeting dietary reference intakes of macronutrients, fruit, vegetables, and fish. The percentage of participants with intakes below the DRIs was highest for fiber (96%). The percentages of Dutch food bank recipients with intakes above the optimal intake of protein and total fat were 98 and 29%, respectively (Table 3). For saturated fat and trans-fat, the percentage of participants with intakes above the DRIs were 88 and 12%, respectively, which can be considered as unhealthy.

**Table 3:** Percentages (95% CIs) of Dutch food bank recipients (*n* = 167), the DNFCS-all (*n* = 1933), and the DNFCS-low-SES (*n* = 312) not meeting dietary reference intakes of macronutrients, fruit, vegetables, and fish

	DRI	Food bank (95% CI)	DNFCS-all (95% CI)	DNFCS-low-SES (95% CI)
Carbohydrates total (en%)	40–70^a^			
Lower bound	40	13.3 (5.9–18.6)^b,c^	25.5 (22.8–27.9)	26.0 (19.9–32.4)
Upper bound	70	0.1 (0–0.2)	0.0 (0–0.0)	0 (0–0.0)
Mono- and disaccharides (en%)	–	–	–	–
Polysaccharides (en%)	–	–	–	–
Protein total (en%)	8–10^a^			
Lower bound	8	0.01 (0–0.23)	0.0 (0.0–0.01)	0 (0–0.02)
Upper bound	10	98.2 (97.3–100)	99.6 (99.4–99.8)	99.7 (99.2–100)
Fat total (en%)	20–40^d^			
Lower bound	20	0.12 (0–0.46)	0.04 (0.01–0.08)	0.02 (0–0.12)
Upper bound	40	25.8 (5.4–44.1)	8.3 (6.4–10.7)	13.5 (8.2–20.0)
Fat monounsaturated (en%)	–	–	–	–
Fat polyunsaturated (en%)	12^e^	1.04 (0–1.24)	0.18 (0.06–0.4)	0.1 (0–0.37)
Fat saturated (en%)	10^f^	88.1 (84.1–98.9)	91.4 (89.5–95.2)	91.7 (88.4–96.2)
Fat trans (en%)	1^f^	12.3 (2.8–16.1)^b^	1.7 (0.8–2.6)	1.9 (0.3–3.9)
Dietary fiber (g)	30–40^g^			
Lower bound	30	95.9 (94.2–99)	94.0 (92.0–95.4)	96.5 (93.6–98.1)
Upper bound	40	0.6 (0–1.0)	0.2 (0.1–0.3)	0.1 (0.0–0.3)
Fruit (g)	200^g^	97.6 (94.3–100)^b^	89.2 (85.1–90)	96.1 (92.6–98.5)
Vegetables (g)	200^g^	86.6 (81.4–95.1)	93.5 (89.5–95)	97.4 (93–100)
Fish (g)	34^h^	99.3 (92.5–100)^b^	86.3 (83.5–89.8)	91.7 (84.8–96.9)

DNFCS-all: data from the Dutch National Food Consumption Survey 2007–2010 among 1933 adults in the age range 23–69 years

DNFCS-low-SES: data from the Dutch National Food Consumption Survey 2007–2010 among 312 low educated adults in the age range 23–69 years

*DRI* dietary reference intake

*en%* energy percentage

– Not available

^a^Recommended dietary allowance for men and women in the age ranges 19–30, 31–50, and 51–70 years

^b^Significantly different from the DNFCS-all, determined by comparing the 95% CIs

^c^Significantly different from the DNFCS-low-SES, determined by comparing the 95% CIs

^d^Daily adequate intake for men and women with normal weight, overweight or undesirable weight gain in the age ranges 19–30, 31–50, and 51–70 years. The daily adequate intake for overweight people or people with undesirable weight gain is 20–30/35 en%

^e^Daily tolerable upper intake level for men and women in the age ranges 19–30, 31–50, and 51–70 years

^f^Daily tolerable upper intake level for men and women in the age ranges 19–30, 31–50, and 51–70 years; daily adequate intake = as low as possible

^g^Daily recommendation for men and women in the age ranges 19–30, 31–50, and 51–70 years

^h^Based on the guideline of two time fish per week and the average weight of 120 g for a single portion

For alcohol, over 75% of the Dutch food bank recipients consumed less than or one glass of alcohol per day, and over 10% consumed more than two glasses of alcohol.

Mean intakes of fruit, vegetables, and fish were lower than the dietary guidelines (Table 2). The large majority of the Dutch food bank recipients had a lower intake for fruit (98%), vegetables (87%), and fish (99%) (Table 3).

### Differences in dietary intake between Dutch food bank recipients and DNFCS-all and DNFCS-low-SES

The mean habitual intakes of energy, alcohol, and dietary fiber of Dutch food bank recipients were significantly lower, whereas the proportion of energy intakes derived from carbohydrates, polysaccharides, and trans-fat were significantly higher than those of the DNFCS-all and the DNFCS-low-SES (Table 2). Among the Dutch food bank recipients, the percentage of people with trans-fat intake above the daily tolerable upper level only was significantly higher compared with the DNFCS-all (Table 3).

For Dutch food bank recipients, mean fruit intake was significantly lower compared with the DNFS-all and the DNFCS-low-SES. Dutch food bank recipients’ mean fish intake was significantly lower compared to the DNFCS-all. Mean vegetables intake of Dutch food bank recipients differed significantly neither from the DNFCS-all nor from the DNFCS-low-SES (Table 2).

Percentages of Dutch food bank recipients not meeting the fruit and fish guidelines for a healthy diet were significantly higher compared with the DNFCS-all, but were similar to the DNFCS-low-SES. There was no significant difference in the percentage of people meeting the vegetable guideline (Table 3).

## Discussion

The present study is the first to describe the dietary intake of Dutch food bank recipients and its comparison with the dietary intake of the DNFCS-all and the DNFCS-low-SES. Our study shows that the majority of the Dutch food bank recipients, similar to DNFCS-all and the DNFCS-low-SES, do not meet the dietary reference intakes for saturated fat (en%), dietary fiber (g), fruit (g), vegetables (g), and fish (g). Furthermore, regarding intakes of energy, trans-fat, dietary fiber, fruit, and fish their diet quality is significantly poorer than that of the DNFCS-all and the DNFCS-low-SES. For trans-fat, fruit, and fish, these percentages were significantly higher compared with the representative general sample.

Our results are comparable to those of other studies on dietary intake of food-assistance users: a lower consumption of energy [13], fiber [6, 7, 12], fruit and vegetables [6, 7, 10–12], and seafood [7, 10], compared with the national nutritional guidelines [3, 6, 7, 10, 11, 13], the general population [10], or the low-SES population [7, 11]. In addition, two recently conducted reviews [8, 9], including some of the studies described above, conclude that food-assistance users do not meet dietary recommendations. On the other hand, some differences were found between our study and other studies. In contrast with our study, the study by Jacobs-Starkey et al. [13] showed no differences in energy intake between food-assistance users and the general Quebec population. Furthermore, Leung et al. [7] did not find differences in intakes of energy, and trans-fat between food-assistance users and low-income non-food-assistance users. The differences in results between these two studies and our study might be explained by the small difference in SES between the food-assistance program users and non-users in the two studies. SES differences between the Dutch food bank recipients and both comparison groups were much greater. Moreover, the food system in the study by Leung et al. [7] greatly differs from ours (providing food stamps vs. providing food parcels).

Despite the previously reported high prevalence of food insecurity (72.9%) [4], and the current observation that Dutch food bank recipients do not meet the dietary guidelines for a healthy diet, 56.3% of the participants were (very) satisfied with their current food intake and 47.9% perceived their current food intake as (very) healthy. Therefore, it is important to make the Dutch food bank recipients aware of their poor diet quality as this can contribute to improving their food intake.

In our previous study, we calculated the average weighed provided nutrients, fruit, vegetables, and fish for a single-person food parcel for one single day [1]. If we compare these results with the mean dietary intake of the Dutch food bank recipients from the current study, we see discrepancies in energy (1986 vs. 4744 kcal), fiber (16.5 vs. 49 g), fruit (62.8 vs. 97 g), vegetables (123 vs. 295 g), and fish (8.5 vs. 23 g). We previously showed [1] that 60.6% of the Dutch food bank recipients did not consume all foods from the food parcel because, e.g., the foods are beyond the expiration date. Although the content of food parcels was measured in a different period of time (October 2010–April 2011) than dietary intake was measured (September 2011–February 2012), this might be an explanation for the discrepancy in provided amounts in the food parcel and the actual intake.

In general, Dutch food bank recipients included in our study engage more in unhealthy lifestyle behaviours than the comparison groups. The proportion of smokers (58.1%) was (more than) twice as high compared with the DNFCS-all (26.1%) and the DNFCS-low-SES (29.2%). In addition, severe overweight (37.0%) was much more prevalent than in the DNFCS-all (18.6%) and the DNFCS-low-SES (22.1%). Reported levels of physical activity, however, were comparable. Together with their current poor diet quality, this emphasizes the vulnerability of this group for diet-, body-weight-, and smoking-related diseases.

The results of our study should be interpreted in the context of its strengths and limitations. This study is the first to describe the dietary intake of Dutch food bank recipients and to investigate the differences in dietary intake between Dutch food bank recipients and the DNFCS-all and the DNFCS-low-SES. For this comparison, we used the original data of the DNFCS 2007-2010, which makes the results more accurate. Furthermore, strengths include the methods that we used to analyse dietary intake data. By estimating the habitual dietary intakes instead of mean dietary intakes per participant, we were able to take the between and within person variation into account. Moreover, by collecting dietary intake data from three different weeks, and in more than 60% of the participants on two different weekdays and one weekend day within 1 month, we tried to take the week-to-week variation into account, as well as the fact that dietary intake may vary over the time of the month (e.g., due to payment of salary or social-assistance benefit). Furthermore, previous studies, for instance, in low-income households showed that (several) multiple pass 24-h dietary recalls are considered most accurate to represent the overall energy, carbohydrate, protein, fat, or nutrient intake [18, 34, 35], though underreporting is often observed in all self-reporting of dietary intake [36, 37]. In addition, it is known that people with a higher BMI [36–38], lower education [36], lower income [39], or lower literacy [40] show greater underreporting compared with their counterparts.

A possible limitation of this study is the period of time in which we collected data at the Dutch food banks. We collected data between September 2011 and February 2012, but preferably, we would have collected data throughout a whole year to be able to cover all seasons. It is known that seasonal variation may influence the type and/or quantity of fruit and vegetable intake of adults [41]. Although this variation might be less between autumn and winter as compared to summer and winter, we cannot exclude some influence of season. Furthermore, excluding the food bank recipients with an inadequate command of the Dutch language from our study might have led to a less representative sample of Dutch food bank recipients. At last, for the analyses of dietary intake of the Dutch food bank recipients, the 2010 NEVO database was used, whereas for the analyses of dietary intake of the DNFCS-all and DNFCS-low-SES, the 2011 NEVO database was used. Trans-fat levels of the foods in the 2010 NEVO database are slightly higher, which might have led to an overestimation of trans-fat intake in Dutch food bank recipients.

A consideration for the interpretation of the results of our study is the difference in the definition of low SES between the Dutch food bank recipients and the DNFCS-low-SES. For the Food Bank Study, low level of education was based on less than finished elementary school as highest completed level of education. The DNFCS-low-SES was defined by combining primary and lower vocational education as highest completed level of education, due to the small number of people with finished elementary school only (*n* = 48). Therefore, SES is even lower in the Dutch food bank recipients. This may have contributed to the differences found between Dutch food bank recipients and the DNFCS-low-SES.

Our study provides evidence for an unhealthy diet of Dutch food bank recipients regarding macronutrient, fruit, vegetables, and fish intake. They meet key dietary guidelines less often than the DNFCS-all and the DNFCS-low-SES. Our results highlight the need of improving dietary intake of Dutch food bank recipients. Strategies need to be developed to optimize the dietary intake of Dutch food bank recipients, e.g., distributing information on healthy food intake, adding recipes to the food parcel, or improving the content of the food parcels. The content of the food parcels could be improved by adding more fruit and vegetables, and removing products which are high in saturated fat. Moreover, research is needed to gain insight in micronutrient intake, and to identify the most effective approaches to improve the dietary intake of this vulnerable population subgroup. The latter could be accomplished by assessing the effect of altering the content of the food parcels on dietary intake of Dutch food bank recipients in an intervention study.
